# Depth-distribution patterns and control of soil organic carbon in coastal salt marshes with different plant covers

**DOI:** 10.1038/srep34835

**Published:** 2016-10-06

**Authors:** Junhong Bai, Guangliang Zhang, Qingqing Zhao, Qiongqiong Lu, Jia Jia, Baoshan Cui, Xinhui Liu

**Affiliations:** 1 State Key Laboratory of Water Environment Simulation, School of Environment, Beijing Normal University, Beijing 100875, P.R. China

## Abstract

This study was carried out in three kinds of salt marshes according to the vegetation covers, including *Phragmites australis* salt marsh (PSM), *Suaeda salus* salt marsh (SSM) and *Tamarix chinensis*-*Suaeda salus* salt marsh (TSSM). We applied allometric function, exponential function and logistic function to model the depth distribution of the SOCv and SOCc for each salt marsh, respectively. The results showed that the exponential function fits the depth distribution of the SOCv more well than other two functions. The SOCc can be fitted very well by all three functions for three salt marsh (Adj. R^2^ > 0.99), of which the allometric function was the best one. The mean topsoil concentration factors (TCFs) of three salt marshes were beyond 0.1, which means the SOC enrichment in surface soils due to plant cycling, but TCFs in PSM were significantly higher than those in SSM (P < 0.05). Nearly 30% of SOC was concentrated in the top 20 cm soils. The results of general linear model (GLM) suggested that four soil properties (soil water content, pH, soil salt content and silt+clay) and their interactive effects explained about 80% of the total variation of SOC stock in the top 20 cm soils and the 20–100 cm soil layers.

Coastal salt marshes play an important role in maintaining the balance of atmospheric carbon dioxide and serve as carbon sink with an estimated carbon burial rate of 210 g C/m^2^/yr^1^,. Unlike most upland soils, coastal salt marshes can continuously sequester carbon through the plant production and burial process^2^,^3^,^4^. Therefore, coastal salt marshes are an important blue carbon ecosystem and serve as highly efficient carbon sink.

The soil organic carbon (SOC) in salt marshes can indicate the climate change^5^, and the dynamic changes in SOC storage have a significant impact on the global carbon cycle^6^. However, our current understanding of the SOC quantity and distribution in salt marshes is limited. The SOC stocks in coastal salt marshes could be under- or overestimated as a result of large uncertainties^7^. Therefore, it is essential to make an accurate estimate of the SOC stocks in the coastal salt marshes to quantify the SOC sink capacity of the coastal salt marshes^6^.

Coastal salt marshes are complex ecosystems due to the interactions between fluvial and marine processes. Under the influence of hydrological fluctuations, vegetation succession and burial processes, the SOC in salt marshes typically have a large spatial variation. Previous studies have estimated the SOC stocks on the global^1^,^8^, national^9^,^10^ and regional scales^11^,^12^,^13^, but these estimates have shown large discrepancies. Limited attention has been given to coastal salt marshes with high hydrological fluctuations and large amounts of sediment supplies. Currently, the profile distribution model^14^,^15^,^16^ and multiple regression approach^11^,^12^,^17^, combined geostatistics^14^,^15^ and artificial neural network^18^ techniques are the main methods for estimating the SOC stocks in a selected region. Several models have been applied to describe and extrapolate the SOC content^16^, and the exponential functions might be most widely used in modelling the vertical SOC distribution^14^ due to its mathematical simplicity and its apparent similarity to the SOC decline with the soil depth^15^. However, it remains unknown whether the exponential function could be fitted to the vertical SOC distribution in salt marshes.

The Yellow River Delta (YRD) is the broadest, youngest and most efficiently conserved wetland ecological system in the warm temperate zone of China^19^. Large amounts of sediment inputs from the Loess Plateau caused the delta to expand fast at a rapid rate of ~9.11 km^2^/yr from 1976–2009^20^. According to the results of Yu *et al*.^19^, the SOC density of the YRD ranged from 0.73 kg/m^2^ to 4.25 kg/m^2^ at a depth of 0–30 cm. There were approximately 3.55 × 10^6^ t SOC stored in the YRD. To our knowledge, few studies have reported the SOC stocks at depths of 0–100 cm in salt marshes of the YRD. In addition, vegetation types, via the shoot/root allocations combined with vertical root distributions^16^, can significantly affect the depth distribution of the SOC. Therefore, the study area was divided into three types of salt marshes based on the dominant vegetation covers: the *Phragmites australis* salt marsh (PSM), the *Suaeda salus* salt marsh (SSM) and the *Tamarix chinensis-Suaeda salus* salt marsh (TSSM). The primary objectives of this study were: (1) to simulate the depth distribution patterns of volumetric SOC (SOC_*v*_, kg/m^3^) and cumulative SOC stocks (SOC_*c*_, kg/m^2^) using different mathematic equations in three salt marshes; (2) to predict the SOC stocks (0–80 cm and 0–100 cm depth) using three functions in three salt marshes and to compare the prediction accuracy, and (3) to compare the depth distribution patterns of the SOC under the three vegetation covers and analyse the controls of the SOC stocks at different depth intervals.

## Results and Discussion

### Soil characterization

The summary statistics of the SOC, bulk density (BD), pH, soil salt content (SSC) and soil texture (sand, silt and clay) of all samples (n = 28) in the study area are shown in Table 1. The SOC contents ranged from 0.48 to 8.20 g/kg in the study area, with mean SOC values of 1.65 to 3.96 g/kg. A decreasing trend was observed along the soil profile according to the mean values of SOC, with the exception of the 40–60 cm soil layer, which was more similar to the SSC trend. The soil pH values indicated a weak alkaline environment. A relatively high BD (>1.55 g/cm^3^) could be attributed to the serious compaction and breakdown of the soil structure in coastal areas^21^.

Soil BD and pH exhibited weak variability (Coefficient of Variation (CV), with CV values less than or equal to 10%), whereas SOC and SSC exhibited moderate variability (10% < CV < 100%)^22^ at all depth intervals. For the soil texture, strong variability (CV greater than or equal to 100%) was found in the clay content despite its lower content. The sand and silt also exhibited moderate levels of variability, thus implying the existence of intensive hydrological fluctuations in the study area.

### Modelling the depth distribution of the soil organic carbon

**Figure 1:**
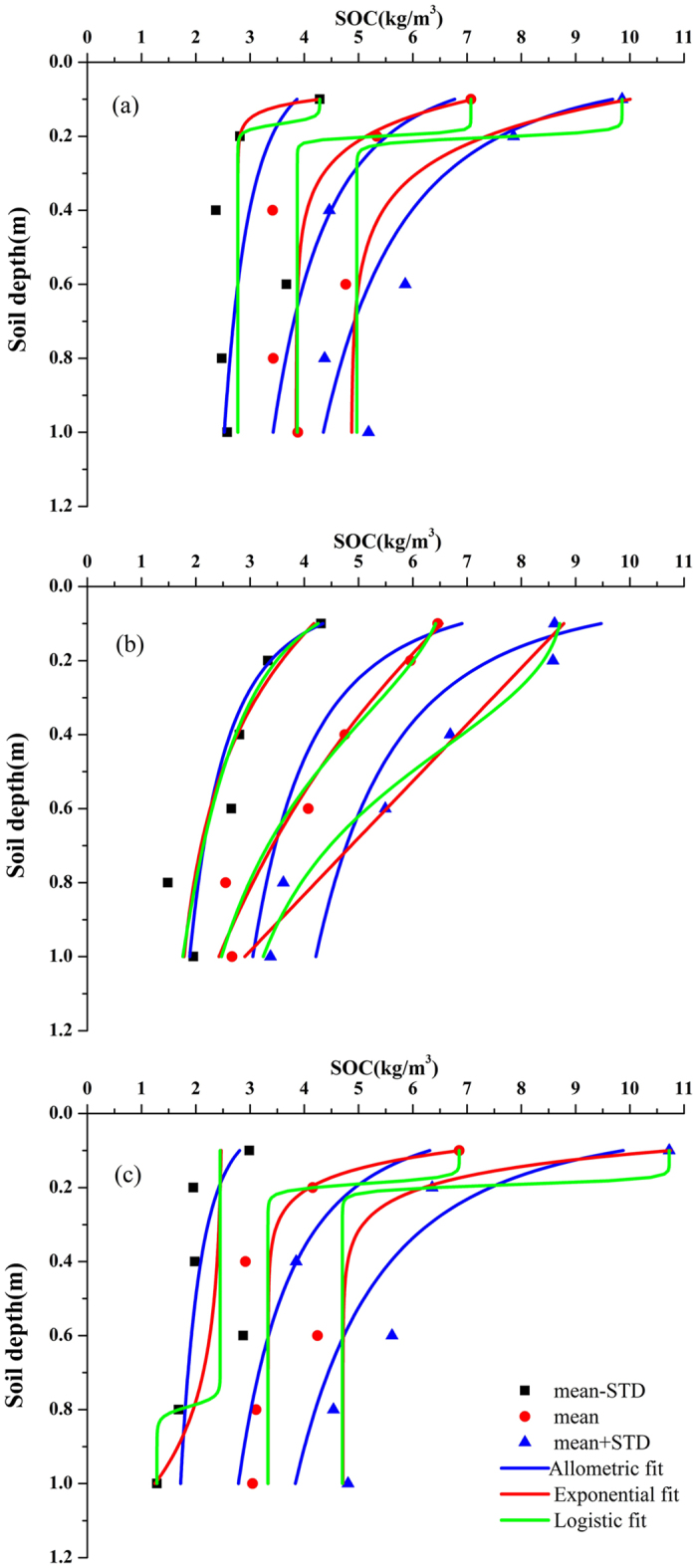
Fitting curves of vertical distribution of volumetric SOC (SOC_*v*_) using three functions in *Phragmites australis* salt marsh (PSM, (**a**)), *Suaeda salus* salt marsh (SSM, (**b**)) and *Tamarix chinensis-Suaeda* salus salt marsh (TSSM, (**c**)).

**Figure 2:**
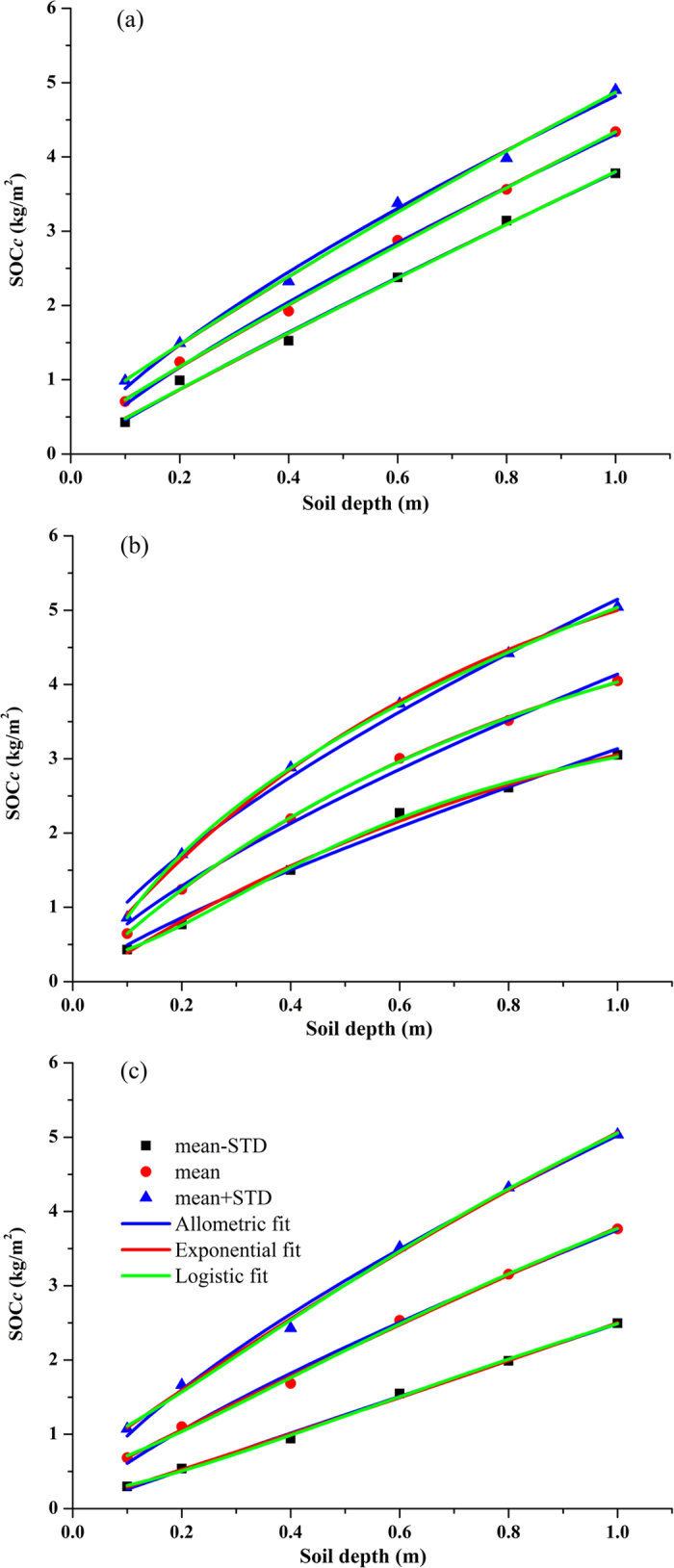
Fitting curves between cumulative SOC stocks (SOC_*c*_) and soil depths using three functions in PSM (**a**), SSM (**b**) and TSSM (**c**).

The data in the calibration data sets were used to model the depth distribution of volumetric SOC (SOC_*v*_, kg/m^3^) and cumulative SOC stocks (SOC_*c*_, kg/m^2^). The results of the fitting using three equations are shown in Figs 1 and 2. The detailed fitting equation and fitting results are listed in Tables 2 and 3. When modelling the depth distribution of the SOC_*v*_, the exponential function showed the best modeling result compared with allometric and logistic functions in spite of its low goodness of fit, with mean Adj. R^2^ = 0.76, 0.95 and 0.82 for PSM, SSM and TSSM, respectively. The decay exponential function has been widely applied to describe the vertical SOC distribution in forestland^22^,^23^,^24^, agricultural land^25^,^26^ and grassland^16^. The exponential function might therefore be used to predict the SOC stocks regardless of the soil type or land use^13^,^15^. In our study, the fitting results indicated that the exponential function is also useful in modelling the vertical distribution of volumetric SOC (kg/m^3^) in coastal salt marshes.

**Figure 3:**
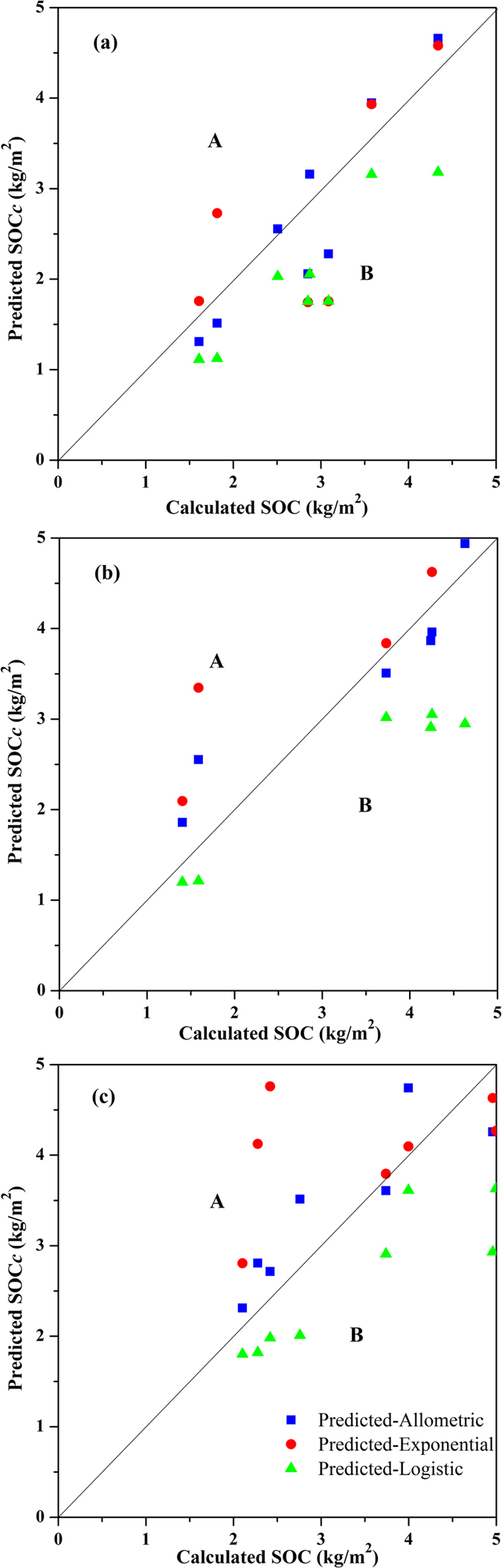
Prediction results obtained using three functions and the relationship between predicted and calculated cumulative SOC stocks (SOC_*c*_) in PSM (**a**), SSM (**b**) and TSSM (**c**). Some unusual predicted values are not shown in this figure. Area A denotes overestimations, and area B denotes underestimations.

Interestingly, the values of SOC_c_ were theoretically equal to the integral values of SOC_*v*_ in terms of the soil depth from 0 cm to the desired soil depth. However, this could produce many errors when using the integral values of SOC_*v*_ given its irregular distribution (Fig. 2) and low goodness of fit (Table 2). Therefore, we calculated the SOC_*c*_ based on the SOC_*v*_ and used three mathematical functions to describe the depth distribution of the SOC_*c*_. When modelling the depth distribution of SOC_*c*_, three equations all showed high goodness of fit (Adj. R^2^ > 0.99) for the three salt marshes (Table 3). Therefore, it is necessary to determine which function would result in lower errors when predicting the SOC stocks among three modelling functions. Figure 3 showed the relationship between the calculated SOC_*c*_ values (0–80 cm and 0–100 cm) and the predicted SOC_*c*_ values using the three functions in the three salt marshes.

### Prediction and validation of the cumulative soil organic carbon stock (SOC_c_)

In many cases, we need to predict the SOC density (kg/m^2^) for a given type of land. This process would be time-consuming and expensive if we measured the SOC values of all samples at different depths. Therefore, it can be helpful to predict the SOC stocks of a desired depth using the SOC_*v*_ content in the surface soils. In our study, we used the SOC_*c*_ of four intervals of upper soil layers (0–10, 0–20, 0–40, and 0–60 cm) in validation data sets to predict the SOC_*c*_ of the other two intervals (0–80 and 0–100 cm). The results of predicted SOC_*c*_ values (0–80 and 0–100 cm) in the three salt marshes are shown in Fig. 3. The data in Table 4 show the validation indices (MPE and RMSE) of the predicted SOC_*c*_ values at the 0–80 and 0–100 cm intervals in the three salt marshes using the three functions.

As shown in Fig. 3, all of the SOC_*c*_ values predicted using logistic function were in area B, which means that the predicted values were smaller than the calculated values. In contrast, almost all of the SOC_*c*_ values predicted using exponential functions were in area A, which indicates that the exponential function may result in larger predicting outcomes. Further, the predicted SOC_*c*_ values using the allometric function were distributed on both sides of an oblique line with a slope of 1, which indicated that the allometric function might be applied to predict the SOC stocks in coastal salt marshes. The validation indices shown in Table 4 confirm the availability of the allometric function. Positive values of MPE indicated that the predicted values were larger than the observed values, and this was reversed for negative MPE values. The lowest absolute values of MPE and RMSE were found in the three salt marshes when using an allometric function. The lower absolute values of MPE and RMSE suggest that the allometric function fitting method of SOC_*c*_ produces fewer errors when predicting the SOC stocks^14^.

The results of the single sample *t*-test shown in Table 5 indicate that the slope of the regression line of predicted and calculated SOC_*c*_ based on the allometric and exponential functions is not significantly different than 1, thus suggesting acceptable predictions of SOC_*c*_ compared to the logistic function. However, the slopes based on the allometric and exponential functions are significantly different than 1, which suggests that the SOC stocks are either under- or overpredicted^14^.

### Topsoil concentration factors (TCFs) of the three salt marshes

**Figure 4:**
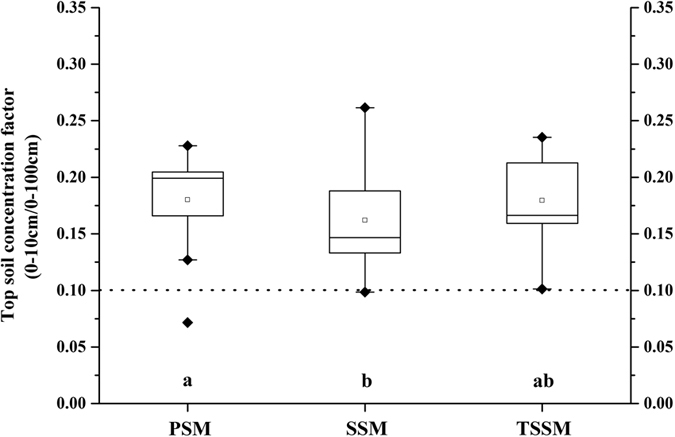
Topsoil concentration factors (TCFs, 0–10 cm/0–100 cm) of volumetric SOC values (SOC_*v*_, kg/m^2^) in the three salt marshes (^ab^Values with different letters represent significant differences between different salt marshes, *P* < 0.05).

Topsoil concentration factors (TCFs) can be used to evaluate the effects of plant cycling on biogeochemical elements^27^. The box plots shown in Fig. 4 illustrate the TCFs in the three salt marshes. The mean TCFs (0–10 cm/0–100 cm) of the three salt marshes were all greater than 0.1, which indicates the presence of an SOC enrichment in the surface soil (0–10 cm) due to plant cycling. The TCF values of the PSM were significantly higher than those in the SSM and slightly higher than those in the TSSM (*P* < 0.05). These differences should be ascribed to the different vegetation covers in the salt marshes.

**Figure 5:**
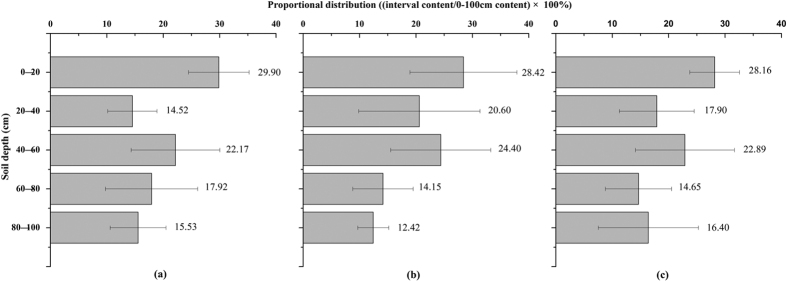
Profiles of volumetric SOC (SOC_*v*_) distributions in PSM (**a**), SSM (**b**) and TSSM (**c**). (mean + SD). There were not significant differences among the three salt marshes in the same layers (*P* > 0.05) based on one-way ANOVA.

Plant characteristics such as tissue stoichiometry, biomass cycling rates, above- and below-ground allocation, root distribution, and maximum rooting depth^27^ might play an important role in the distribution patterns of SOC. Figure 5 illustrates the proportional distribution of the SOC content ((soil layer/0–100 cm) × 100%) in the PSM, SSM and TSSM. The SOC content in the surface soil layer (0–20 cm) was relatively higher than that in the other layers in the three salt marshes. There was a decreasing trend for SOC, with the exception of the 40–60 cm soil layer. The unexpected peak of SOC in the 40–60 cm layer may be explained by the following two reasons. On one hand, it may be associated with the high silt and clay contents in this layer (Table 1). Zinn *et al*.^28^ also demonstrated that the SOC content was directly and linearly correlated with the combined clay + silt (but not the clay alone) content for all depths at the 0–1 m interval. On the other hand, this unexpected peak could be explained by downward migrating of soil organic carbon by leaching and microbial activities^29^,^30^ in the surface soils.

Many researches have shown that plant production is a major SOC input to soil in arid and semi-arid ecosystems^16^,^31^,^32^. However, with the exception of plant litter input, the organic carbon burial due to the sediment accumulation^33^ and tidal flooding input play an important role in the SOC budgets and depth distribution patterns of the SOC for coastal salt marshes.

### Relationships between the SOC stocks and soil properties at different depth intervals

**Figure 6:**
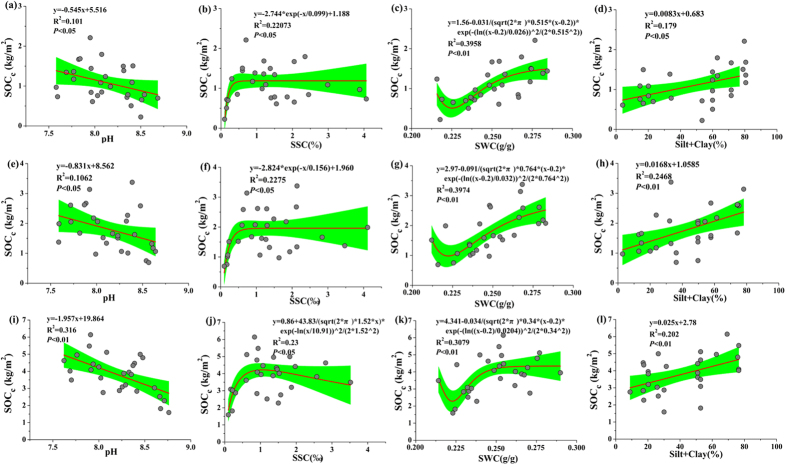
Relationships between the cumulative SOC stocks (SOC_*v*_) at different intervals (i.e., 0–20 cm, 0–40 cm and 0–100 cm) and soil properties. The green shaded areas show the mean 95% confidence intervals.

The relationships between the cumulative SOC stocks (SOC_*c*_) at different depth intervals (0–20, 0–40 and 0–100 cm) and soil properties such as pH, SSC, soil water content (SWC) and silt + clay content are shown in Fig. 6. Positive liner relationships were found between SOC_*c*_ at different depth intervals and silt +clay (Fig. 6(d,h,l)). Similarly, a SOC = a + b(silt + clay) function was proposed by Zinn *et al*.^28^ to describe the relationship between the SOC and clay+silt, which was also available for any depth at the 0–1 m interval. As reported by Yang *et al*.^31^, the soil texture influences the SOC storage in two ways. First, it increases the clay and silt contents and reduces microbial decomposition by stabilizing the SOC and decreasing C leaching, thus leading to an accumulation of SOC. Second, increasing the clay and silt contents stimulate plant production by increasing the water holding capacity and thereby increasing C inputs to soil. The SOC_*c*_ would reach a relatively stable value based on the relationships between SSC, SWC and SOC_*c*_ (Fig. 6(b,c,f,g,j,k)). In terms of the SSC, a low level of salinity may increase the microbial decomposition rates by stimulating extracellular enzyme activity and enhancing the bacterial abundance^34^, which eventually causes, at least in part, a decreased level of SOC accumulation, accretion and carbon sequestration rates in tidal areas. This explanation is consistent with our study results, which indicate that low SOC stock levels can be found in salt marshes with low SSC levels (SSC < 5‰) (Fig. 6b,f,j). A high SSC content may reduce the microbial activity to affect the SOC decomposition^34^,^35^, and the accumulation of salts in the root zone may have an adverse effect on plant growth by decreasing the availability of water to the plants and affecting the metabolism due to specific ion toxicity and ion imbalances^36^. Consequently, a moderate salinity level might be beneficial for the carbon sequestration of coastal salt marshes.

Our study showed that nearly 30% of the SOC (based on the 0–100 cm reserve) was concentrated in the soil surface (0–20 cm) of the coastal salt marshes of the YRD (Fig. 5). Furthermore, the soil surface area is more obviously affected by processes such as weathering, plant litter decomposition and water flooding than are deep soil layers (20–100 cm in this study). Therefore, a GLM was applied to the soil surface and the deep soil layers to analyse the relationships between the SOC_*c*_ and soil physicochemical properties and to identify the contributing factors, respectively. The results of the GLM suggested that the four selected soil properties (i.e., SWC, pH, SSC and silt + clay) explained 81.23% and 79.02% of the total variation of SOC stock in the top 20 cm and 20–100 cm layers, respectively (Table 6). SWC explained the largest proportion (41.64%) of the SOC stock variation, whereas the pH explained approximately 11.3% of the variation for the 0–20 cm layer. However, the pH explained the largest proportion (32.54%) of the variation in the SOC stock, and SWC only explained 9.42% of the variation for the 20–80 cm soil layer. It is important to note that the controlling factors described above are interactively affected. For example, increased soil pH can limit the binding capacity of clay compounds, leading to decreased organic matter (e.g., humic acid) sorption in soil^34^,^37^. Furthermore, weather factors (e.g., rain) and hydrological fluctuation (e.g., tidal flooding and flow-sediment regulation projects) would also significantly change the water content (SWC) and soil salinity (SSC) in surface soils and other soil physicochemical properties^38^ in this area.

## Conclusions

The depth distributions of the volumetric SOC contents (SOC_*v*_, kg/m^3^) and the cumulative SOC stocks (SOC_*c*_, kg/m^2^) were modelled using allometric, exponential and logistic functions in three salt marshes with different plant covers. The modelling data were based on our sampling results in representative coastal salt marshes of the Yellow River Delta in China. The decay exponential function can better fit the depth distribution of SOC_*v*_ in the coastal salt marshes than the other two functions despite its low goodness of fit, which is widely used in terrestrial ecosystems for the estimation of SOC stock. The depth distribution of the SOC_*c*_ can be fitted very well by three functions for each salt marsh (Adj. R^2^ > 0.99), however, the values of MPE and RMSE, and *t*-test results indicated more accurate predictions of SOC_*c*_ in the top 100 cm soils using the allometric function in comparison to both exponential and logistic functions. Vegetation cover types can affect the depth distribution pattern of SOC by plant cycling, root distribution changes and above- and below-ground allocation differences according to the topsoil concentration factor analysis. The general linear model analysis showed that the pH and soil moisture (SWC) were the main controlling factors of the SOC storage in the study area. The co-effects of environmental factors such as the pH, soil moisture, soil salt and soil texture on the SOC distribution and the quantitative mathematic functions among them will require further research in coastal salt marshes.

## Materials and Methods

### Study area

The study was conducted in the newly formed salt marshes of the Yellow River Delta, China. This region is characterized by a warm-temperate and continental monsoon climate, with an annual mean precipitation of 640 mm and an annual mean evaporation of 1962 mm. The air temperature varies from −23.3 to 41.9 °C and the annual mean value is 12.3 °C^39^. The Yellow River water and sediment discharges have significant seasonal variability, and more than 60% of the river water and sediment^40^ is discharged during the flooding season (from June to July). The soil is typical Fluvisols, which is derived from the upstream of the Loess Plateau^41^. Dominant vegetation types in the study area are *Phragmites australis*, *Suaeda salus* and *Tamarix chinensis.* PSM was mainly distributed along the Yellow River banks. The distribution area of SSM was near the coastline. The TSSM was in the ecotone of *Phragmites australis* and *Suaeda salus.* Some *Suaeda saluses* were growing under the cover of *Tamarix chinensis*.

### Sample collection and analysis

We identified 10, 8 and 10 profiles in PSM, SSM and TSSM, respectively. The soil samples were collected from soil pits at depths of 0–10, 10–20, 20–40, 40–60, 60–80, and 80–100 cm. In total, 60, 48 and 60 samples were obtained from the PSM, SSM and TSSM, respectively, and the soil samples were used for the determination of the SOC, pH, soil salt content (SSC) and soil texture. All samples were sealed in polyethylene bags and brought to the laboratory, then air dried at room temperature for three weeks. All air-dried samples were sieved through a 2-mm nylon sieve to remove coarse debris and stones, then ground with a pestle and mortar until all particles passed a 0.149-mm nylon sieve for the determination of their soil chemical properties (i.e., SOC, pH and SSC). Additionally, in each profile, a single 4.8-cm diameter soil core was collected from each depth interval. The soil core was oven dried at 105 °C for 24 h and weighed for the determination of its bulk density (BD) and soil water content (SWC).

A Hach pH meter (Hach Company, Loveland, CO, USA) was used to measure the soil pH (soil:water = 1:5). SSC was determined in the supernatant of 1:5 soil-water mixtures using a salinity meter (VWR Scientific, West Chester, PA, USA). The SOC mass concentration (g/kg) was measured using the bichromate oxidation method^42^. Soil particle size analysis was conducted on a laser particle size analyzer (Microtrac S3500, America). All samples were analysed in triplicate.

### Data processing

The SOC data were divided into calibration and validation data sets. In the PSM, there were 36 calibration data points and 24 validation data points. Similarly, there were 30 calibration data points and 18 validation data points for the SSM, 36 calibration data points and 24 validation data points for the TSSM, respectively. We applied allometric, exponential and logistic functions to model the depth distribution of the SOCv and SOCc for each salt marsh. The formulas of the three functions are shown as follows:


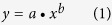



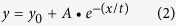



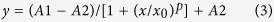


where eq. (1) is the allometric function, eq. (2) is the exponential function, and eq. (3) is the logistic function. The volumetric SOC (SOC_*v*_, kg/m^3^) can be obtained by multiplying the SOC mass concentration (g/kg) by the soil BD (kg/m^3^) (Eq. (4)):


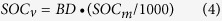


where SOCv is the volumetric SOC (kg/m^3^), BD is the bulk density (kg/m^3^) of the soil sample, and SOC_*m*_ is the SOC mass concentration (g/kg) of the sample.

For a given profile, we assumed that the SOC is distributed uniformly in a given depth interval. Therefore, the SOC stock (kg/m^2^) in this interval is the product of the volumetric SOC and interval depth (m) (Equation (5)). We define the numbers 1, 2, 3, 4, 5, and 6 as denoting the depths of 0–10, 10–20, 20–40, 40–60, 60–80, and 80–100 cm, respectively. Thus, the SOC_*c*_ from the surface (0 cm) to a givendepth is the sum of SOC stock in soil layers, which is calculated by eq. (6). Thus, for a profile, the SOC_*c*_ at the depth of 0 to 60 cm is calculated by the sum of the SOC stocks in the 0–10, 10–20, 20–40, and 40–60 cm layers.


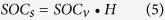



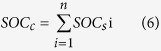



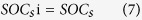


where SOC_*s*_ is the SOC stock of a given depth interval, H is the interval depth, and SOC_*c*_ is the cumulative SOC stock in a desired depth. In our study, n is from 1 to 6, SOC_*s*_i is the SOC stock for the *i*_*th*_ layer, and SOC_*s*_i is equal to SOC_*s*_ (Eq. (7)) when eq. (5) is used to calculate the SOC stock in the *i*_*th*_ layer.

Three different functions were used to model the depth distribution of the volumetric SOC (SOC_*v*_) and the cumulative SOC stock (SOC_*c*_). In the process of modelling the depth distribution of SOC_*v*_, the allometric, exponential and logistic functions were fitted to describe the depth distribution of SOC_*v*_ for each profile using a nonlinear least squares procedure in the calibration data sets. The fitting depth was from the surface (0 cm) to 100 cm. In contrast, when modelling the depth distribution of SOC_*c*_, three functions were fitted to describe the depth distribution of SOC_*c*_ in the individual soil profiles.

### Validation of predicted cumulative SOC stocks

In this study, we used the SOC_*c*_ of four intervals of the layers (0–10, 0–20, 0–40, and 0–60 cm) in validation data sets to predict the SOC_*c*_ values of the other two intervals (0–80 and 0–100 cm). Three equations were all applied for this prediction. By calculating different validation indices, such as, the mean predictive error (MPE) and root mean square error (RMSE), we can compare the predictive veracity among the three equations. The formulas of MPE and RMSE are shown below.


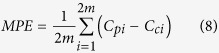



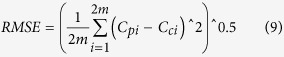


where C_*pi*_ is the predicted value of the cumulative SOC stock, C_*ci*_ is the calculated value of the cumulative SOC stock based on measured values, and m is the number of samples used to validate the model in each salt marsh. The MPE represents the bias of the prediction and the RMSE represents the average error of the prediction^14^. These values should approach zero for an optimal prediction. The performance of an extrapolated function was evaluated by the regression analysis of predicted and observed values through comparisons with a 1:1 relationship^16^,^43^. Furthermore, the *t*-test was used to test the hypothesis that the slope of the regression line between the calculated and predicted SOC stocks (SOC_*c*_) equals 1^14^,^44^.

### Topsoil concentration factors (TCFs)

In this study, TCFs were employed to evaluate the effects of different vegetation on the SOC in salt marshes. If the TCFs (0–10 cm/0–100 cm) were greater than 0.1, the SOC enrichment in the surface soil (0–10 cm) could be attributed to plant cycling.


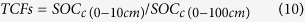


where SOC_*c*(0–10 cm)_ is the cumulative SOC stock in the surface soil (0–10 cm) and SOC_*c*(0–100 cm)_ is the cumulative SOC stock in 0–100 cm soil.

### Statistical analysis

The Origin 8.0 software package was used to model the depth distribution patterns of the SOC_*v*_ and SOC_*c*_ in the PSM, SSM and TSSM, respectively. One-way analysis of variance (ANOVA) was used to test the significant differences of topsoil concentration factors (TCFs) among the three salt marshes, Differences were considered to be significant if *P* < 0.05. A general linear model (GLM) was used to assess the integrative effects of the four individual soil properties (i.e.,pH, soil water content, soil salt content and silt + clay content) on the SOC density (SOC_*v*_ in this study)^31^ at 0–20 cm and 20–100 cm intervals. GLM analysis and one-way ANOVA were performed using the R (R version 3.2.4) for Windows software package.

## Additional Information

**How to cite this article**: Bai, J. *et al*. Depth-distribution patterns and control of soil organic carbon in coastal salt marshes with different plant covers. *Sci. Rep.* **6**, 34835; doi: 10.1038/srep34835 (2016).

## Figures and Tables

**Table 1:** Summary statistics of soil properties at different depth intervals (n = 28).

Soil depth	SOC				BD				pH				SSC			
	Mean (g/kg)	Min (g/kg)	Max (g/kg)	CV (%)	Mean (g/cm^3^)	Min (g/cm^3^)	Max (g/cm^3^)	CV (%)	Mean	Min	Max	CV (%)	Mean (‰)	Min (‰)	Max (‰)	CV (%)
0–10 cm	3.96	1.12	8.20	41.3	1.69	1.28	2.03	10.28	8.05	7.56	8.75	3.73	1.54	0.10	4.86	80.75
10–20 cm	2.62	0.48	5.05	52.67	1.70	1.42	2.03	10.21	8.18	7.58	8.73	4.12	1.24	0.10	3.56	71.45
20–40 cm	1.92	0.68	4.46	48.94	1.42	1.92	1.71	9.53	8.24	7.58	8.83	4.69	1.23	0.10	4.21	78.65
40–60 cm	2.46	0.66	4.83	39.11	1.74	1.32	2.02	11.57	8.22	7.62	8.82	4.21	1.31	0.10	3.19	62.82
60–80 cm	1.68	0.56	3.63	47.58	1.75	1.45	1.93	8.80	8.26	7.40	8.90	4.32	1.09	0.10	2.66	56.88
80–100 cm	1.65	0.56	3.71	51.92	1.76	1.45	2.01	9.68	8.30	7.48	9.20	4.79	1.04	0.10	2.93	61.08
**Soil depth**	**Sand**				**Silt**				**Clay**							
	**Mean (%)**	**Min (%)**	**Max (%)**	**CV (%)**	**Mean (%)**	**Min (%)**	**Max (%)**	**CV (%)**	**Mean (%)**	**Min (%)**	**Max (%)**	**CV (%)**				
0–10 cm	41.34	16.60	91.95	66.68	55.77	8.05	78.43	47.28	2.88	0.00	8.59	78.72				
10–20 cm	60.05	16.87	100.00	43.86	40.61	0.00	79.24	63.83	1.09	0.00	6.99	170.82				
20–40 cm	69.05	24.99	99.58	30.91	32.06	0.42	71.48	69.21	1.17	0.00	4.97	8.61				
40–60 cm	48.99	10.11	96.70	47.65	51.01	3.30	79.88	42.66	3.57	0.00	15.86	14.61				
60–80 cm	63.84	26.81	100.00	41.57	39.00	0.00	97.66	71.32	1.06	0.00	2.89	189.40				
80–100 cm	67.58	22.15	100.00	48.88	34.87	0.00	97.77	96.30	1.43	0.00	3.14	148.28				

CV: Coefficient of Variation.

**Table 2:** Model parameters and fitting results of volumetric SOC (SOC_*v*_) values using mathematic equations for the three salt marshes.

Salt marsh	Function	Allometric Y = a*x^b	Exponential Y = y0+A*exp(−x/t)	Logistic Y = (A1−A2)/(1+(x/x0)^p)+A2
PSM	mean	SOC_*v*_ = 3.43*H^−0.30	SOC_*v*_ = 3.85 + 8.31*exp(−H/0.107)	SOC_*v*_ = (7.07−3.87)/(1 + (H/0.20)^35.75) + 3.87
		R^2^ = 0.72931	R^2^ = 0.76365	R^2^ = 0.69706
	mean-STD	SOC_*v*_ = 2.52*H^−0.19	SOC_*v*_ = 2.77 + 60.77*exp(−H/0.027)	SOC_*v*_ = (4.28−2.77)/(1 + (H/0.17^20.22) + 2.77
		R^2^ = 0.27039	R^2^ = 0.38739	R^2^ = 0.0811
	mean + STD	SOC_*v*_ = 4.35*H^−0.35	SOC_*v*_ = 4.87 + 10.61*exp(−H/0.138)	SOC_*v*_ = (9.86−4.97)/(1 + (H/0.20)^26.45) + 4.97
		R^2^ = 0.80683	R^2^ = 0.8334	R^2^ = 0.84607
SSM	mean	SOC_*v*_ = 3.05*H^−0.35	SOC_*v*_ = −0.36 + 7.63*exp(−H/0.99)	SOC_*v*_ = (6.57−1.12)/(1+(H/0.58)^2.03)+1.12
		R^2^ = 0.8434	R^2^ = 0.95147	R^2^ = 0.93606
	mean-STD	SOC_*v*_ = 1.89*H^−0.36	SOC_*v*_ = 1.51 + 3.43*exp(−H/0.40)	SOC_*v*_ = (32.90 + 13.63)/(1+(H/9.62)^0.10)-13.63
		R^2^ = 0.87492	R^2^ = 0.83537	R^2^ = 0.77208
	mean + STD	SOC_*v*_ = 4.21*H^−0.35	SOC_*v*_ = −81008.37 + 810097.81*exp(−H/124043.19)	SOC_*v*_ = (8.76−2.11)/(1+(H/0.56)^2.76)+2.11
		R^2^ = 0.78358	R^2^ = 0.9483	R^2^ = 0.96931
TSSM	mean	SOC_*v*_ = 2.79*H^−0.36	SOC_*v*_ = 3.33 + 16.22*exp(−H/0.066)	SOC_*v*_ = (6.86−3.33)/(1+(H/0.19)^21.97)+3.33
		R^2^ = 0.69743	R^2^ = 0.82459	R^2^ = 0.74399
	mean-STD	SOC_*v*_ = 1.72*H^−0.21	SOC_*v*_ = 2.50+(−0.02)*exp(−H/(−0.24))	SOC_*v*_ = (2.45−1.29)/(1 + (H/0.79)^63.32)+1.29
		R^2^ = 0.23265	R^2^ = 0.15737	R^2^ = −0.03645
	mean + STD	SOC_*v*_ = 3.84*H^−0.41	SOC_*v*_ = 4.71 + 24.53*exp(−H/0.071)	SOC_*v*_ = (10.73−4.70)/(1+(H/0.19)^25.74)+4.70
		R^2^ = 0.72665	R^2^ = 0.90381	R^2^ = 0.87026

STD: standard deviation.

**Table 3:** Model parameters and fitting results of cumulative SOC (SOC_*c*_) values, as determined using mathematic equations for the three salt marshes.

Salt marsh	Function	Allometric Y = a*x^b	Exponential Y = y0+A*exp(−x/t)	Logistic Y = (A1−A2)/(1+(x/x0)^p)+A2
PSM	mean	SOC_*c*_ = 4.30*H^0.81	SOC_*c*_ = 23.35+(−23.05)*exp(−H/5.20)	SOC_*c*_ = (0.23–679.4)/(1+(H/264.3)^0.91)+679.4
		R^2^ = 0.99673	R^2^ = 0.99696	R^2^ = 0.99551
	mean−STD	SOC_*c*_ = 3.79*H^0.92	SOC_*c*_ = 35.18+(−35.08)*exp(−H/8.96)	SOC_*c*_ = (0.05–1990.7)/(1+(H/758.7)^0.95)+1990.7
		R^2^ = 0.99522	R^2^ = 0.99369	R^2^ = 0.9906
	mean + STD	SOC_*c*_ = 4.82*H^0.74	SOC_*c*_ = 19.38+(−18.88)*exp(−H/3.80)	SOC_*c*_ = (0.45–62.5)/(1+(H/15.14)^0.95)+62.5
		R^2^ = 0.99448	R^2^ = 0.99559	R^2^ = 0.99341
SSM	mean	SOC_*c*_ = 4.14*H^0.73	SOC_*c*_ = 5.71+(−5.72)*exp(−H/0.82)	SOC_*c*_ = (−0.001–8.188)/(1+(H/x1.03)^1.05)+8.188
		R^2^ = 0.99235	R^2^ = 0.9916	R^2^ = 0.99426
	mean−STD	SOC_*c*_ = 3.14*H^0.80	SOC_*c*_ = 4.85+(−4.94)*exp(−H/0.988)	SOC_*c*_ = (0.276–4.19)/(1+(H/0.61)^1.75)+4.19
		R^2^ = 0.98734	R^2^ = 0.99353	R^2^ = 0.99883
	mean + STD	SOC_*c*_ = 5.15*H^0.68	SOC_*c*_ = 6.67+(−6.61)*exp(−H/0.728)	SOC_*c*_ = (−1.30–51.62)/(1+(H/52.30)^0.50)+51.62
		R^2^ = 0.99211	R^2^ = 0.99838	R^2^ = 0.99993
TSSM	mean	SOC_*c*_ = 3.75*H^0.79	SOC_*c*_ = 20.36+(−20.05)*exp(−H/5.26)	SOC_*c*_ = (0.49–10.14)/(1+(H/1.65)^1.33)+10.14
		R^2^ = 0.99524	R^2^ = 0.99653	R^2^ = 0.99544
	mean−STD	SOC_*c*_ = 2.49*H^0.98	SOC_*c*_ = −22.71+22.77*exp(−H/(–9.81))	SOC_*c*_ = (0.19–7.87)/(1+(H/1.82)^1.43)+7.87
		R^2^ = 0.99687	R^2^ = 0.99661	R^2^ = 0.99598
	mean + STD	SOC_*c*_ = 5.02*H^0.71	SOC_*c*_ = 15.71+(−15.15)*exp(−H/2.83)	SOC_*c*_ = (0.77–12.62)/(1+(H/1.56)^1.28)+12.62
		R^2^ = 0.99462	R^2^ = 0.99613	R^2^ = 0.9946

STD: standard deviation.

**Table 4:** Validation indices of cumulative SOC stocks (SOC_*c*_) using three functions in three salt marshes.

Salt marsh	Index	Allometric function	Exponential function	Logistic function
PSM	MPE	−0.15	1.97	−0.81
	RMSE	0.47	4.94	0.87
SSM	MPE	0.14	4.03	−0.92
	RMSE	0.50	7.40	1.06
TSSM	MPE	0.28	1.61	−0.82
	RMSE	0.54	3.33	0.99

MPE: mean predictive error, kg/m^2^. RMSE: root mean square error, kg/m^2^.

**Table 5:** Results of a *t*-test used to test the hypothesis that the slope of the regression line equals 1 between calculated and predicted SOC_*c*_ values using three functions.

Salt marsh	PSM(2 m = 8)		SSM(2 m = 6)		TSSM(2 m = 8)	
Function	*t* statistic	*P* value	*t* statistic	*P* value	*t* statistic	*P* value
Allometric	−1.283	0.240	1.138	0.307	2.176	0.066
Exponential decrease	1.223	0.261	1.845	0.124	1.632	0.147
Logistic	−7.944	0.000	−7.818	0.001	−6.639	0.000

2 m is the number of predicted values (cumulative SOC stock in 0–0.8 m and 0–1 m) used for a single sample *t*-test (confidence interval = 95%). m is the number of soil samples in the validation data sets of each type of salt marsh.

**Table 6:** Summary of the results obtained from a general linear model (GLM), showing the integrative effects of the soil water content (SWC), pH, soil salinity content (SSC) and soil texture (silt + clay) on soil organic carbon (SOC) stocks in surface (0–20 cm) and deep (20–100 cm) layers.

Source(0–20 cm)	df	Parameters	SE	MS	SS(%)	Source(20–100 cm)	df	Parameters	SE	MS	SS(%)
SWC	1	−328.5**	90.99	0.47625***	41.64	SWC	1	−182.8	117.1	0.046087*	9.42
pH	1	−8.834**	2.474	0.12924**	11.30	pH	1	−4.935	3.115	0.159209***	32.54
SSC	1	−25.00*	11.31	0.0016	0.14	SSC	1	7.733	3.686	0.008865	1.81
Silt.Clay	1	0.1909*	0.06963	0.02865	2.51	Silt.Clay	1	−0.6013	0.6509	0.014703	3.00
SWC × pH	1	40.97**	11.15	0.01207	1.06	SWC × pH	1	22.61	14.13	0.016776	3.43
SWC×SSC	1	121.7*	50.34	0.01856	1.62	SWC × SSC	1	−9.083	4.870	0.004218	0.86
pH × SSC	1	3.119*	1.418	0.06719*	5.87	pH × SSC	1	−0.6463	0.3724	0.07188**	14.69
pH × Silt.Clay	1	−0.02329*	0.008423	0.09024*	7.89	SWC × Silt.Clay	1	3.149	2.903	0.018718	3.83
SSC × Silt.Clay	1	−0.09336	0.04943	0.00004	0.00	pH × Silt.Clay	1	0.07363	0.07744	0.000104	0.02
SWC × pH × SSC	1	−15.16*	6.332	0.05666	4.95	SSC × Silt.Clay	1	−0.1844*	0.08258	0.010461	2.14
pH × SSC × Silt.Clay	1	0.01166	0.006138	0.04847	4.24	SWC × pH × Silt.Clay	1	−0.3829	0.3466	0.000171	0.03
Residuals	16			0.01342	18.77	SWC × SSC × Silt.Clay	1	0.1721	0.1354	0.009405	1.92
						pH × SSC × Silt.Clay	1	0.01688	0.008957	0.026051	5.32
						Residuals	14			0.007332	20.98

***P < 0.001; **P < 0.01; *P < 0.05; *P* < 0.1. df, degree of freedom; SE, standard errors; MS, mean squares (*F*-test); SS, proportion of variances explained by the variable; Silt. Clay, silt+clay SOC density was log_10_-transformed before analysis. The parameters of the GLM model and their standard errors (SE) were also presented (*t*-test).
